# Supplementary material for: Development and Characterization of an Ex Vivo Testing Platform for Evaluating Automated Central Vascular Access Device Performance
Source: J Pers Med. 2022 Aug 5;12(8):1287. doi: 10.3390/jpm12081287 (PMC9410168; doi:10.3390/jpm12081287)
Supplement: Supplementary file 1 [file jpm-12-01287-s001.zip › jpm-1833460-supplementary.pdf]

Supplementary Information

Supplementary Table S1. Summary of Device Specific Criteria. Note these were not evaluated in this manuscript as they are device specific, and likely independent of the model being used.

Device Specific Criteria
Failure detection
Scan breadth
Catheter size/needle gauge compatibility
Provisions for sanitation of device and patient
Dislodgement detection
Auto-tuning of probe or other sensors
Guidewire guidance
Training time recommended by vendor
Average time to attach and activate system
Personnel proficiency at different experience/training levels
Ingress Protection Rating
Light emission
Patient movement/vibration
Noise produced
Ambient temperature
Atmospheric pressure
Battery life
Overall device footprint
Weight
